# Weekly External Load Correlation in Season Microcycles with Game Running Performance and Training Quantification in Elite Young Soccer Players

**DOI:** 10.3390/s24144523

**Published:** 2024-07-12

**Authors:** Vasileios Kanaras, Yiannis Michailidis, Athanasios Mandroukas, Andreas Stafylidis, Lazaros Vardakis, Angelos E. Kyranoudis, Kosmas Christoulas, Ioannis Gissis, Thomas I. Metaxas

**Affiliations:** Laboratory of Evaluation of Human Biological Performance, Department of Physical Education and Sports Sciences, Aristotle University of Thessaloniki, 54124 Thessaloniki, Greece; vasiliskanaras8@gmail.com (V.K.); amandrou@phed.auth.gr (A.M.); astafylidis@phed.auth.gr (A.S.); vardakisl@phed.auth.gr (L.V.); akyra@phed.auth.gr (A.E.K.); kchristo@phed.auth.gr (K.C.); igkisis@phed-sr.auth.gr (I.G.); tommet@phed.auth.gr (T.I.M.)

**Keywords:** young, football, external load volume, performance

## Abstract

The purpose of this study was to (a) correlate the weekly external training load with the game running performance in season microcycles and (b) specify the optimal training/game ratio of the weekly external load in elite youth soccer players. The total distance (TD), the high-speed running distance (HSRD) (19.8–25.2 km/h), the ZONE6 distance (>25.2 km/h), the acceleration (ACC) (≥+2 m/s^2^), and the deceleration (DEC) (≥−2 m/s^2^) were monitored with global positioning system (GPS) technology throughout 18 microcycles and official games. TD had a very high positive correlation average (r = 0.820, *p* = 0.001), the HSRD had a high positive correlation average (r = 0.658, *p* = 0.001), the ZONE6 distance and DEC had a moderate positive correlation average ((r = 0.473, *p* = 0.001) and (r = 0.478, *p* = 0.001), respectively), and the ACC had a low positive correlation average (r = 0.364, *p* = 0.001) between microcycles and games. Regarding the training/game ratio, the HSRD showed statistically significant differences between ratios 1.43 and 2.60 (*p* = 0.012, *p* ≤ 0.05), the ACC between ratios 2.42 and 4.45 (*p* = 0.050, *p* ≤ 0.05) and ratios 3.29 and 4.45 (*p* = 0.046, *p* ≤ 0.05), and the DEC between ratios 2.28 and 3.94 (*p* = 0.034, *p* ≤ 0.05). Considering the correlation between weekly training and game external load, high weekly training TD values correspond to higher game values, whereas HSRD, ZONE6 distance, ACC, and DEC, which determine training intensity, should be trained in a specific volume. Training/game ratios of 1.43, 2.42 to 3.29, and 2.28 to 3.11 seem to be optimal for HSRD, ACC, and DEC weekly training, respectively.

## 1. Introduction

Recent global positioning system (GPS) development in football has improved the understanding of the sport’s physical and physiological aspects by accurately recording daily workloads during training sessions and games [1]. The training load (TL) is the input variable used to elicit the desired training response [2] and is differentiated into internal and external loads [3], both of which represent the cumulative exposure of each athlete to training sessions and games [3]. The internal load is the psycho-physiological stress of the player’s body during exercise, while the external load refers to all the activities that a football player can perform during training or a game [4] and can be measured using a GPS, which has proven to be a valid and reliable tool [5]. The use of this technology can provide data about distances covered in different velocities and bouts of acceleration (ACC) and deceleration (DEC) at different intensities and running velocities during training sessions and games [6]. Based on scientific evidence, the total distance (TD) covered, the distance covered at high-speed running measured between 19.8 and 25.2 km/h (HSRD), the distance covered at sprint running (SPR) measured over 25.2 km/h, the specific maximal speed (e.g., to record in the game), the number of ACC ≥ +3 m/s^2^, and the number DEC ≤ −3 m/s^2^ seem to be relevant GPS parameters to monitor the external TL in professional soccer [6,7,8,9].

The quantification of training and games represents an important procedure for adjusting the training stimuli provided to players according to game demands [10]. The monitoring and quantification of GPS data can help coaches and strength and conditioning specialists to better periodize the training process and increase players’ and, subsequently, the team’s performance [11]. Several studies [12,13,14,15,16] have quantified the weekly external training and game load over a whole competitive season. Stevens et al. [11] described the weekly external TL in professional Dutch soccer players. The information about the external load showed that the duration of the training, TD, estimated energy expenditure, time >90% HRmax, run, medium and high ACC, medium and high DEC, and high power were higher on game day (GD)-4, and only the HSR (19.8 m/s–25.2 m/s) was higher on GD-3 in a four-day training microcycle (weekly training). In the same study, the cumulative weekly training load (one game and four subsequent training sessions) was expressed as the number of games that equaled this load for each variable. The total weekly load was higher for the ACC variables (3.1–3.9 games) compared to running (2.5 games) and HSR (2.1 games).

Despite the quantification of the weekly external load described in most studies, there is a growing interest in the correlation of the weekly training running performance with the game running performance or outcome. Clemente et al. (2019) [17] recorded a low positive correlation for TD, HSR, SD, ACC, and DEC between weekly training and game load, while Modric et al. (2021) [18] recorded a low positive correlation for TD and moderate positive correlations for HSR, SD, and high-intensity ACC and DEC. In the same study, the authors recorded the relationship between the weekly external load and game outcomes and described how the weekly load could influence game results, showing that there was a strong negative correlation between the weekly external load and the game result, meaning that there were more possibilities for the team to win when the number of weekly external loads was lower. In the same study, there was a positive correlation between some external training and game variables, such as the total distance covered, the distance covered in low and moderate zones, the high-intensity distance covered, and the high-intensity number of accelerations and decelerations, and it was highlighted that higher values of these variables in weekly training may be positively correlated with the values of the same variables in subsequent games. Similar findings were noticed in a study [19] of a professional soccer team. In this study, it was observed that the team was more successful at winning games when the cumulative weekly training load was significantly lower.

For many decades, professional soccer clubs have invested large amounts of money in their youth academies and development process [20]. The monitoring and analysis of training and game data are fundamental for the development of young players’ physical skills. Using these data, physical coaches can properly design their training plan to develop players’ physical capacity in the (i) short and (ii) long term, preparing them for the next game and age groups, respectively. There are several studies on time–motion analyses in youth players describing the weekly external [21] and game loads [20,22], but there is a lack of literature on the correlation between the two in young soccer players. Modric et al. (2021) [18] described the influence of the weekly external load on the game’s result, showing that the chances of positive match outcomes were greater in weeks when the team participated in fewer training sessions and, consequently, had lower values of external training load. For these age groups, where the initial target is player development and the game result is secondary, the influence of the weekly external running load on game running performance should be examined.

Moreover, there is a lack of information on TL quantity in the youth soccer players’ microcycle. Several studies [7,17] have recorded training/game ratios in professional soccer players at 3 dW (3 days a week), 4 dW, and 5 dW. Rave et al. (2020) [23] proposed using GPS data to analyze, prescribe, and control TL in elite professional soccer. In this study, the proposed weekly TL according to game data was TD × 3.2, HSR and sprint distance × 2.5 and × 1.5 game distance, respectively, and DEC × 3.4 and ACC × 3.4 the game numbers. To date, only the study by Dios-Alvarez et al. (2021) described training/game ratios for young soccer players, where the following 6 dW training/game ratios were recorded: 2.1 for TD, 1.2 for HSR, 0.9 for sprinting distance, 3.5 for ACC (>3 m/s^2^), and 3.3 for DEC (<−3 m/s^2^).

For this reason, the purpose of this study was to investigate the potential correlations between training load and game performance variables in young soccer players, as indicated by variations in training/game ratio differences across different categories (CATs) of total distance covered, high-speed running distance, ZONE6 distance, accelerations, and decelerations. These correlations aim to provide insights into optimizing training strategies to enhance specific aspects of game performance.

## 2. Materials and Methods

### 2.1. Study Design

Data collected from an elite youth male soccer team during the competitive 2019–2020 season were analyzed. Eighteen training microcycles and official games were monitored, each consisting of five training sessions and one official game. The training program was designed by the team’s head and physical coach, with no intervention from the scientific group. The external load of the training units was correlated with the external load of the game occurring at the end of the same microcycle.

Regarding training volume quantification, the training/game ratio was calculated for each variable in all 18 microcycles, with 3 microcycle categories (CATs) created for each variable accordingly. The training/game ratio CATs are shown in Table 1 below. 

### 2.2. Subjects

The group characteristics (age, height, weight, and fat mass) of the twenty elite young male soccer players who participated in this study are shown in Table 2. The subjects played in a U17 elite soccer team and competed in the U17 Greek Superleague during the 2019–2020 season, finishing first (out of 15 teams). Player data fulfilling the following criteria were analyzed: (a) 90 min played in the game, (b) all player positions except for the goalkeeper, and (c) participation in all microcycle training units. All the subjects were informed of the procedures, methods, benefits, objectives, and possible risks involved in this study before giving their written informed consent. This study was approved by the ethics committee of the Aristotle University of Thessaloniki (97/2021).

### 2.3. Training Content 

A typical microcycle consisting of five training sessions and one official game is presented in Table 3. Game day (GD) +1 was always a day off for the team. According to the technical staff’s microcycle periodization, GD-4 targeted high-speed running distance (19.8–25.2 km/h) and sprint distance (>25.2 km/h) exposure, using tactical games with offensive/defensive transition, while GD-3 targeted power, with many accelerations and decelerations, using small-sided games. Furthermore, GD+2 targeted hypertrophy or maximum strength using resistance training, while GD−2 focused on tactical content and GD-1 on reaction and offensive/defensive set pieces. Before each official game, players undertook a standardized 20 min warm-up, consisting of 2 min of free passing in couples, 8 min of running exercises and dynamic stretching, a 6 min (3 × 2′) small-sided game (4 vs. 4 + 2 jogger players), and 3 min finalization and closing with short-distance sprint and acceleration.

### 2.4. Data Collection

External load data were monitored and collected daily for 18 training microcycles and official games using the Apex global positioning system (18 Hz; Apex, STATSports, Newry, Northern Ireland). The players wore a sports vest with a pocket on the back (between the scapulae), where the devices were placed. This equipment was previously used by Beato et al. and validated as reliable to determine movement patterns [24]. The measurements commenced precisely at the start of each half, as verified by the timestamps in the CSV files. No outliers were detected in the timing data for each half. Apart from these adjustments, the objective data from the STATSports APEX GNSS tracking system remained unaltered. Additionally, for data management and storage efficiency, we anonymized the data, cleaned up column names, and converted the raw data from a CSV to an SPSS format for analyses. These transformations were performed solely for data handling purposes, ensuring the integrity of the original data throughout the study. 

The following variables were assessed: total distance (TD) covered in training (TOTALDISTANCE_TRAINING) and game (TOTALDISTANCE_GAME), high-speed running distance (HSRD) covered in training (HSRD_TRAINING) and game (HSRD_GAME), ZONE6 distance covered in training (ZONE6_TRAINING) and game (ZONE6_GAME), number of accelerations (ACCs) performed during training (ACC_TRAINING) and game (ACC_GAME), and number of decelerations (DECs) performed during training (DEC_TRAINING) and game (DEC_GAME). HSRD and ZONE6 were used to describe the distance covered between 19.8 and 25.2 km/h and over 25.2 km/h, respectively. Furthermore, only ACC ≥ +2 m/s^2^ and DEC ≥ −2 m/s^2^ were analyzed in this study. 

### 2.5. Hypotheses and Statistical Analysis

Based on the study objectives, the hypotheses were as follows:

1. The weekly training load (e.g., total distance (TD), high-speed running distance (HSRD), distance covered in ZONE6, accelerations, and decelerations) during training sessions has a significant correlation with the running performance during games in elite young soccer players.

2. The training/game ratios for the weekly load (e.g., total distance (TD), high-speed running distance (HSRD), distance covered in ZONE6, accelerations, and decelerations) exhibit statistically significant differences across the defined categories (CAT1, CAT2, and CAT3).

Data are presented as the mean ± SD. The Shapiro–Wilk test was used to check the normal distribution of each sample variable, and, once confirmed for all variables besides the ZONE6 distance, it allowed us to use parametric statistical methods. The Mann–Whitney U test was performed for independent samples, and Spearman’s correlation coefficient test was used to determine the correlation between the weekly and game external load in all variables, with *p* < 0.05 as the level of significance.

The Kruskal–Wallis test was used to find the differences between the three training/game ratio MIC categories for all variables, with *p* < 0.05 denoting significance. SPSS version 28.0 was used for all the analyses (SPSS, Inc., Chicago, IL, USA).

## 3. Results

### 3.1. Correlation between Weekly Training Load and Game Performance

The correlations between the training and game load for each variable are presented in Table 4. The TD showed a high positive correlation between the training and game load in MIC1, MIC2, MIC8, MIC13, and MIC16 (0.6 ≤ r ≤ 0.79) and a very high positive correlation in the remaining microcycles (0.8 ≤ r ≤ 1.0), with an average correlation coefficient of r = 0.820 and *p* = 0.001.

HSRD showed a low positive correlation between training and game load in MIC6 (0.2 ≤ r ≤ 0.39) [25]; a moderate positive correlation in MIC8 and MIC16 (0.4 ≤ r ≤ 0.59); a high positive correlation in MIC1, MIC4, MIC5, MIC10, MIC14, and MIC18 (0.6 ≤ r ≤ 0.79); a very high positive correlation in MIC3, MIC9, MIC11, MIC12, MIC13, MIC15, and MIC17 (0.8 ≤ r ≤ 1.0); and no correlation in MIC2 and MIC7 (*p* > 0.05). The average correlation coefficient was r = 0.658, *p* = 0.001.

For the ZONE6 distance, there was a low positive correlation between training and game load in MIC6 (0.2 ≤ r ≤ 0.39), a moderate positive correlation in MIC1, MIC3, MIC4, MIC5, MIC9, MIC10, MIC13, MIC14, MIC15, and MIC17 (0.4 ≤ r ≤ 0.59), a high positive correlation in MIC11, MIC12, and MIC18 (0.6 ≤ r ≤ 0.79), and no correlation in MIC2, MIC7, MIC8, and MIC16 (*p* > 0.05). The average correlation coefficient was r = 0.473, *p* = 0.001.

ACC showed a low positive correlation between training and game load in MIC7 and MIC13 (0.2 ≤ r ≤ 0.39), a moderate positive correlation in MIC2, MIC4, MIC5, MIC10, MIC14, MIC15, and MIC18 (0.4 ≤ r ≤ 0.59); a high positive correlation in MIC3, MIC11, and MIC9 (0.6 ≤ r ≤ 0.79); and no correlation in MIC1, MIC6, MIC8, MIC12, MIC16, and MIC17 (*p* > 0.05). The average correlation coefficient was r = 0.364, *p* = 0.001.

For DEC, there was a low positive correlation between training and game load in MIC8 and MIC12 (0.2 ≤ r ≤ 0.39); a moderate positive correlation in MIC2, MIC3, MIC4, MIC5, MIC10, MIC13, MIC14, MIC15, and MIC16 (0.4 ≤ r ≤ 0.59); a high positive correlation in MIC7, MIC9, MIC11 and MIC18 (0.6 ≤ r ≤ 0.79); a very high positive correlation in MIC1 (0.8 ≤ r ≤ 1.0); and no correlation in MIC6 and MIC17 (*p* > 0.05). The average correlation coefficient was r = 0.478, *p* = 0.001.

### 3.2. Differences between Training/Game Ratios

**Figure 1:**
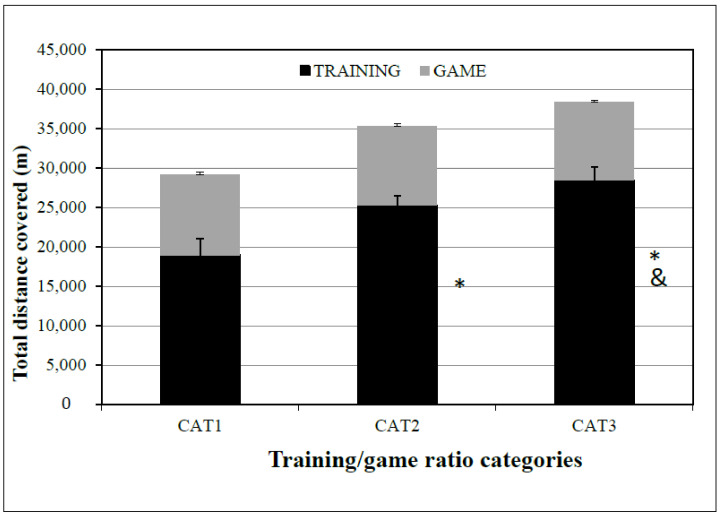
TD differences between CAT1, CAT2, and CAT3 in training and game. TD = total distance covered; CAT1 = microcycles with average TD training/game ratio R = 1.86; CAT2 = microcycles with average total distance training/game ratio R = 2.51; CAT3 = microcycles with average total distance training/game ratio R = 2.90. * Difference from CAT1 TD TRAINING, *p* ≤ 0.05, and difference from CAT2 TD TRAINING, *p* ≤ 0.05. & Differences from CAT2 TD TRAINING, *p* ≤ 0.05.

Regarding the TD training/game ratio differences, the TOTALDISTANCE_TRAINING distance (m) was 18,963 ± 2064, 25,292 ± 1239, and 28,428 ± 1697 for CAT1, CAT2, and CAT3, respectively, with statistically significant differences between the following pairs: CAT1–CAT2 (*p* = 0.005), CAT1–CAT3 (*p* = 0.022), and CAT2–CAT3 (*p* = 0.022). The TOTALDISTANCE_GAME distance (m) was 10,171 ± 418, 10,053 ± 277, and 9917 ± 297 for CAT1, CAT2, and CAT3, respectively, but there were no statistically significant differences between the three categories: CAT1–CAT2 (*p* = 0.762), CAT1–CAT3 (*p* = 0.383), and CAT2–CAT3 (*p* = 0.730) (Figure 1).

**Figure 2:**
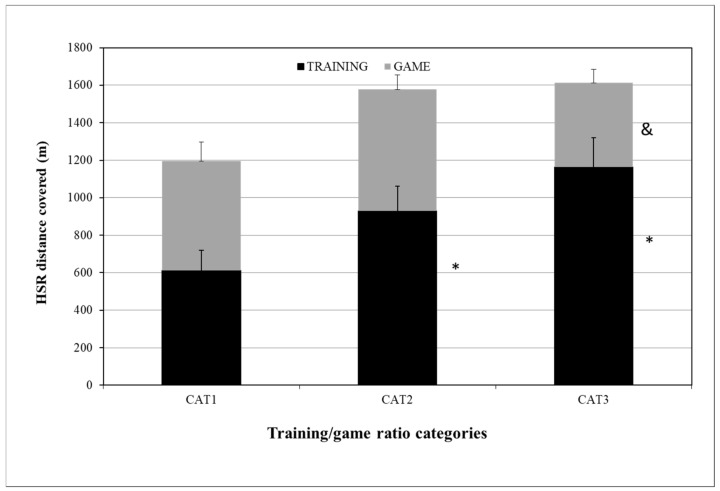
HSRD differences between CAT1, CAT2, CAT3 in training and game. HSRD = distance covered in high-speed running between 19.8 and 25.2 km/h; CAT1 = microcycles with average HSRD training/game ratio R = 1.05; CAT2 = microcycles with average HSRD training/game ratio R = 1.43; CAT3 = microcycles with average HSRD training/game ratio R = 2.60. * Difference from CAT1 HSRD TRAINING and difference from CAT2 HSRD GAME, *p* ≤ 0.05. & Differences from CAT2 HSRD GAME, *p* ≤ 0.05.

For HSRD training/game ratio differences, the HSRD_TRAINING distance (m) was 613 ± 106, 930 ± 131, and 1165 ± 155 for CAT1, CAT2, and CAT3, respectively, with statistically significant differences between CAT1–CAT2 (*p* = 0.012) and CAT1–CAT3 (*p* = 0.017). The HSRD_GAME distance (m) was 584 ± 101, 647 ± 79, and 448 ± 72 for CAT1, CAT2, and CAT3, respectively, with statistically significant differences between CAT1 and CAT3 (*p* = 0.012) (Figure 2).

**Figure 3:**
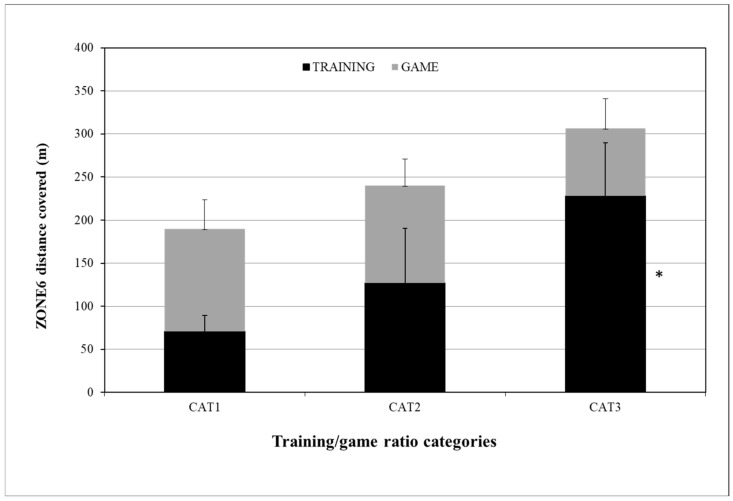
ZONE6 distance differences between CAT1, CAT2, and CAT3 in training and game. ZONE6 distance = distance covered in running speed over 25.2 km/h; CAT1 = microcycles with average ZONE6 distance training/game ratio R = 0.59; CAT2 = microcycles with average ZONE6 distance training/game ratio R = 1.13; CAT3= microcycles with average ZONE6 distance training/game ratio R = 2.91. * Difference from CAT1 ZONE6 TRAINING, *p* ≤ 0.05.

For the ZONE6 training/game ratio differences, the ZONE6_TRAINING distance (m) was 71 ± 19, 127 ± 63, and 228 ± 62 for CAT1, CAT2, and CAT3, respectively, with statistically significant differences only between CAT1 and CAT3 (*p* = 0.011). The ZONE6_GAME distance (m) was 119 ± 34, 113 ± 31, and 79 ± 34 for CAT1, CAT2, and CAT3, respectively, with no statistically significant differences between them (Figure 3).

**Figure 4:**
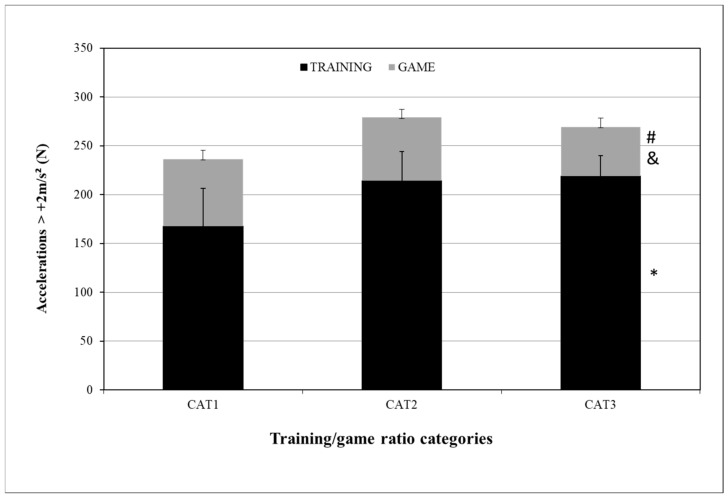
ACC differences between CAT1, CAT2, and CAT3 in training and game. ACC = number of accelerations ≥ +2 m/s^2^; CAT1 = microcycles with average ACC training/game ratio R = 2.42; CAT2 = microcycles with average ACC training/game ratio R = 3.29; CAT3 = microcycles with average ACC training/game ratio R = 4.45. * Difference from CAT1 ACC TRAINING. # Difference from CAT1 ACC GAME and difference from CAT2 ACC GAME, *p* ≤ 0.05. & Differences from CAT2 ACC GAME, *p* ≤ 0.05.

For the ACC training/game ratio differences, the ACC_TRAINING numbers (N) were 167 ± 39, 214 ± 30, and 219 ± 21 for CAT1, CAT2, and CAT3, respectively, with statistically significant differences between CAT1 and CAT3 (*p* = 0.050). The ACC_GAME values (N) were 69 ± 9, 65 ± 8, and 50 ± 9 for CAT1, CAT2, and CAT3, respectively, with statistically significant differences between CAT1–CAT3 (*p* = 0.050) and CAT2–CAT3 (*p* = 0.046) (Figure 4).

**Figure 5:**
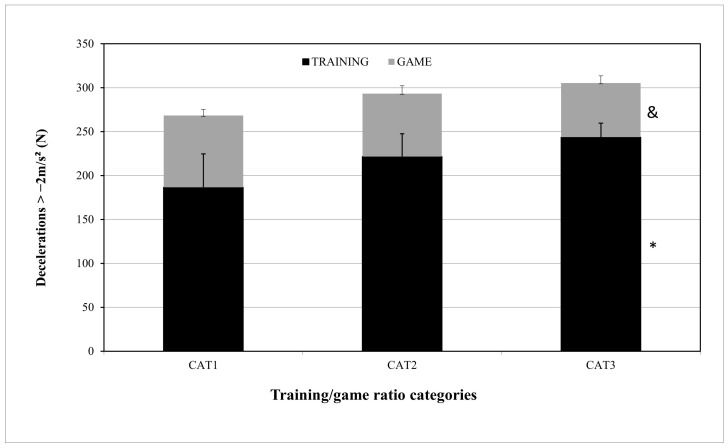
DEC differences between CAT1, CAT2, and CAT3 in training and game. DEC = number of decelerations ≥ −2 m/s^2^; CAT1 = microcycles with average DEC training/game ratio R = 2.28; CAT2 = microcycles with average DEC training/game ratio R = 3.11; CAT3 = microcycles with average DEC training/game ratio R = 3.94. * Difference from CAT1 DEC TRAINING and difference from CAT1 DEC GAME, *p* ≤ 0.05. & Differences from CAT1 DEC GAME, *p* ≤ 0.05.

For the DEC training/game ratio differences, the DEC_TRAINING numbers (N) were 187 ± 38, 222 ± 26, and 244 ± 17 for CAT1, CAT2, and CAT3, respectively, with statistically significant differences between CAT1 and CAT3 (*p* = 0.034). The DEC_GAME numbers (N) were 82 ± 7, 89 ± 40, and 62 ± 8 for CAT1, CAT2, and CAT3, respectively, with statistically significant differences between CAT1 and CAT3 (*p* = 0.034) (Figure 5).

## 4. Discussion

This study has two significant findings: the correlation between training and game external load for variables TD, referring to the training volume, and HSRD, ZONE6, ACC+2, and DEC-2, determining the training and game intensity, and the influence of these variables’ training volume on elite youth players’ game running performance.

Our study results showed an average very high positive correlation (r = 0.820, *p* = 0.001) between training and game TD for all microcycles, suggesting that, if these values are high during the training microcycle, there might be a positive correlation with the TD values in the following game. This is in accordance with a previous study [18] highlighting the positive correlation of the total distance covered between training and game load, mentioning that higher weekly training values could be positively correlated with higher game values, although the study’s correlation between TD training and game load values was much lower than in our study (r = 0.25, *p* < 0.05). Nevertheless, our results contrast those of Clemente et al. (2019) [16], who found no correlation (r = 0.030, *p* = 0.691) between the weekly accumulated external load and the game demands. Modric et al. (2021) [18] highlighted that higher values of high-intensity covered distance and high-intensity ACC and DEC in weekly training can lead to higher game values of the same variables. Regarding the HSRD in our study, there was an average high positive correlation (r = 0.658, *p* = 0.001) between weekly training and game load, in accordance with Modric et al. (2021) [18] (r = 0.48, *p* < 0.05) but contrasting Clemente et al. (2019)’s study [16], where HSRD showed a very low positive correlation (r = 0.153, *p* = 0.042). Regarding the ACC, our results are somewhat in contrast with those of Modric et al. (2021) (r = 0.43, *p* ≤ 0.05). In our study, there was an average low positive correlation (r = 0.364, *p* = 0.001), like Clemente et al. (2019) [16], who noted a low positive correlation between weekly training and game load values for high-intensity ACC (r = 0.292, *p* = 0.001). The DEC values in our study showed an average moderate positive correlation (r = 0.478, *p* = 0.001), while Modric et al. (2021) [18] and Clemente et al. (2019) [16] found a low positive correlation between weekly training and game load for high-intensity DEC r = 0.39, *p* < 0.05, and r = 0.236, *p* = 0.002, respectively. Furthermore, for the ZONE6 values, referring to the sprinting distance covered (>25.2 km/h), there was an average moderate positive correlation (r = 0.473, *p* = 0.001) in our study, aligned with Modric et al. (2021)’s [18] findings (r = 0.51, *p* < 0.05). However, Clemente et al. (2019) [16] found no correlation (r = 0.125, *p* = 0.098) between weekly training and game load.

Considering these results, it seems that, when the factor intensity is outside the training equation, training running distance at low and moderate speeds (<19.8 km/h) can improve the same values in game running distance. Compared to total distance, the variables that determine intensity (e.g., high-intensity running distance >19.8 km/h, sprint distance >25.2 km/h, high-intensity accelerations and decelerations) show different and lower correlations between training and game. It appears that these variables’ microcycle training quantity values can be correlated with the game running performance. Accordingly, the second part of our study, discussed in the following section, ascertained the optimal microcycle training quantity of these variables. 

The total distance training/game ratios were 1.86, 2.51, and 2.90, as mentioned above, with no statistically significant TOTALDISTANCE_GAME differences among them. When the microcycle TD training/game ratio was r = 1.86, the players ran 120 and ~250 m more during the game than at ratios 2.51 and/or 2.90, respectively. Previous studies on professional soccer players noted that the training/game ratio for total distance ranged from 1.87 to 3.1: Stevens et al. (2017) [11] found 3.1 and 2.6 training/game ratios for starters and non-starters, respectively, in a microcycle with four training sessions and one game; Clemente et al. (2019) [17] found a 2.8 total distance training/game ratio in a 4 dW microcycle; and Modri et al. (2021) [18] found a 1.87 total distance training/game ratio among professional soccer players. In a study on elite youth soccer players, like in our study, Dios-Alvarez et al. (2021) [26] recorded a 2.1 total distance training/game ratio, with a weekly total distance of 20,615 m and a total game distance of 9800 m in a 6 dW microcycle. These results are close to our study results, according to which the total distance covered in meters was slightly higher when the training/game ratio was r = 1.86, with no statistically significant differences. 

Regarding HSRD coverage, the training/game ratios were 1.05, 1.43, and 2.60, respectively. In HSRD_GAME, there were statistically significant differences between CAT2 and CAT3 (*p* = 0.012) that were not observed between CAT1 and CAT3 (*p* = 0.058). When the HSRD microcycle training/game ratio was r = 1.43, the players ran more distance at high speeds (19.8–25.2 km/h) during the game, totaling 647 ± 79 m, while, with ratios of 1.05 and 2.60, they ran 584 ± 100 m and 448 ± 72 m, respectively. Stevens et al. (2017) [11] noted a 2.1 training/game ratio in a 4 dW, while Clemente et al. (2019) [16] recorded HSR training/game ratios of 1.1, 2.0, and 2.3 in 3 dW, 4 dW, and 5 dW, respectively. Furthermore, Modric et al. (2021) [18] found a 0.92 HSR training/game ratio among professional soccer players. Previous findings on the HSR training/game ratio among professional soccer players are different from our study results, possibly due to training and game intensities greater than in youth soccer, resulting in a ratio of approximately 2.0. Our results are very close to those of a previous study on elite youth soccer players [26] that recorded a 1.2 HSR training/game ratio in 6 dW, with the optimal training/game ratio in our study being close to 1.43 for the distance covered by the players during the game. Although there were statistically significant differences in game distance only between ratios of 1.43 and 2.60 (200 m), the difference between 1.0 and 1.43 ratios, which was around 64 m, is important from a soccer aspect, as it can play a decisive role in a game’s outcome. 

For the ZONE6 distance, the training/game ratios were 0.59, 1.13, and 2.9, with no significant ZONE6_GAME differences among the three categories. Despite the absence of any significant difference, when the training/game ratio was 0.59, the players covered more distance in the sprint zone (119.29 ± 33.70) than at ratios of 1.13 and 2.91. Previous studies [11,17] on professional soccer players revealed that the 4 dW microcycle sprinting distance was 87 m and 80.9 m. Dios-Alvarez et al. (2021) [26] recorded weekly and game sprinting distances of 205 m and 220 m, respectively, in a 6 dW microcycle among elite youth soccer players, with a training/game ratio of 0.9, while, in our study, these distances ranged from 71 to 228 m and 79 to 119 m, respectively. As previously mentioned, in our study, the microcycle training/game ratio of 0.59 allowed the players to cover more distance in the game—specifically, 6.5 and ~40 m more than ratios of 1.13 and 2.91. Although there were no statistically significant differences between the game sprint distance ratio categories, from a soccer aspect, this 40 m difference in sprinting may be crucial for the game’s outcome. Considering youth soccer players’ long-term improvement in their sprinting ability, a training program with a 3.0 microcycle training/game ratio seems appropriate for players’ game running performance. Certainly, this requires good coaching staff organization in the periodization, taking into consideration players’ acute chronic workload to avoid any injuries.

High-intensity acceleration and deceleration are two variables that have been increasingly examined by researchers. With the coaching staff’s use of small-sided games in the microcycles, accelerations and decelerations have the greatest training/game ratios of all microcycle variables [17,18]. In our study, the ACC training/game ratios were 2.42, 3.29, and 4.45, respectively. In ACC_GAME, there were statistically significant differences between CAT1 and CAT3 (*p* = 0.050) and CAT2 and CAT3 (*p* = 0.046), meaning that, with a microcycle ACC training/game ratio ranging from 2.42 to 3.29, players performed greater accelerations of +2 m/s^2^ than at a training/game ratio of 4.45. Previous studies [11,17,18] conducted among professional soccer players recorded high-intensity acceleration training/game ratios of 3.9, 3.5, and 2.01. Dios-Alvarez et al.’s (2021) [26] study of elite youth soccer players recorded, in 6 dW, an ACC training/game ratio of 3.5, which is not similar but very close to our study results. The training/game ratios for DEC were 2.28, 3.11, and 3.94, respectively. For DEC_GAME, there were statistically significant differences between CAT1 and CAT3 (*p* = 0.034). When the microcycle DEC training/game ratio was 2.28, the players performed more decelerations of −2 m/s^2^ in the game than at ratios of 3.11 and 3.94. Considering that there were no differences between the ratios of 2.28 and 3.11, the optimal DEC training/game ratio could range from 2.28 to 3.11. Previous studies among professional soccer players recorded different training/game ratios: Clemente et al. (2019) [16] recorded DEC training/game ratios of 1.6, 2.8, and 3.4 in 3 dW, 4 dW, and 5 dW, respectively, while Stevens et al. (2017) [11] and Modric et al. (2021) [18] recorded DEC training/game ratios of 3.3 and 1.47, respectively. In another study [26] among elite youth soccer players, the 6 dW DEC training/game ratio was 3.3. The optimal DEC training/game ratio in our study is lower than the ratios recorded in previous studies, possibly due to different age groups (professional vs. youth) [11,17,18] and fewer microcycle training units (5 dW vs. 6 dW) [26]. 

## 5. Conclusions

In conclusion, while the association between professional soccer players’ weekly external load with game running performance and/or results has been examined by previous studies [11,17,18], our study examined the correlation between young soccer players’ weekly training load and game running performance. The results showed a very high positive average correlation (r = 0.820, *p* = 0.001) for TD, a high positive average correlation (r = 0.658, *p* = 0.001) for HSRD, moderate positive average correlations ((r = 0.473, *p* = 0.001) and (r = 0.478, *p* = 0.001)) for the ZONE6 distance and DEC, respectively, and a low positive average correlation (r = 0.364, *p* = 0.001) for ACC. The average positive correlation for each variable suggests that the weekly training load may positively influence players’ game performance: TD training values, which correspond to aerobic capacity, might have a positive influence on TD game values, while HSRD, ZONE6 distance, ACC, and DEC training values, corresponding to anaerobic capacity and power, might influence their respective game values to a lesser extent, depending on these variables’ microcycle training volume.

Considering the above, we examined the training/game ratios of TD, HSRD, ZONE6, ACC, and DEC, comparing differences to reach the optimal ratio for each variable. The TD microcycle training/game ratio at which players ran more distance during the game was 1.86, without any statistically significant differences from the other two recorded ratios (2.51 and 2.90). The optimal HSRD microcycle training/game ratio was 1.43, with a statistically significant difference from the 2.60 ratio (*p* = 0.012) and a difference in the distance covered during the game of 200 m. There was no significant difference from the 1.05 ratio, but the 64 m difference in the distance covered during the game may be important for the game’s outcome. For the ZONE6 distance, there were no statistically significant differences between the three recorded ratios: from a soccer aspect, ratios of 0.59 and 1.13 had differences of 40 and 34 m in the sprinting distance covered during the game compared to the 2.9 ratio, which could influence the game’s results. Furthermore, the fact that there were no statistically significant differences between the three recorded ratios for ZONE6 can be used by coaches for the long-term training of youth soccer players’ sprinting ability. The optimal ACC and DEC training/game ratios ranged from 2.42 to 3.39 and 2.28 to 3.11, respectively, close to ratios for these variables previously recorded in youth soccer players [26].

Several studies have examined and described youth soccer players’ weekly external load and game running performance [20,21,22]. For instance, Dios-Alvarez, in the first study reporting training/game ratios in young soccer players, examined the external and internal load of each day in a 6 dW training microcycle relative to the game’s external and internal load. Our study first examined the potential correlations between weekly training load and game performance variables in young soccer players and, then, compared different training/game ratios to specify the optimal ratio for the aforementioned variables in a training microcycle, differentiating our contribution from previous similar efforts.

This study has some limitations: (a) all data refer to one U17 elite youth team, so comparisons with other populations should be made with caution; (b) the subject pool was small due to the initial inclusion criteria; and (c) the level of the opponent players and game significance were not taken into consideration. 

## 6. Practical Applications

Considering the positive correlation between training and game load and the differences in game performance between the various training/game ratios, different training strategies should be applied for TD, HSRD, ZONE6 distance, ACC +2 m/s^2^, and DEC −2 m/s^2^. Practitioners and technical staff should consider the training volume for variables that determine training intensity, specifically paying more caution to HSRD, ZONE6 distance, ACC +2 m/s^2^, and DEC −2 m/s^2^, using specific training/game ratios to maximize the following game’s performance. Training contents targeting HSRD and the ZONE6 distance, such as transition games in large spaces, which increase high-speed (19.8–25.2 m/s) and sprint (>25.2 m/s) distance and small-sided games, which increase the number of ACC +2 m/s^2^ and DEC −2 m/s^2^, should be applied during microcycles with the optimal training/game ratios proposed in this study to achieve higher game performance among youth soccer players. 

## Figures and Tables

**Table 1:** Training/game ratio categories for each variable.

Training/Game Ratio Category	TD	HSRD	ZONE6 Distance	ACC +2 m/s^2^	DEC −2 m/s^2^
CAT1	1.86	1.05	0.59	2.42	2.28
CAT2	2.51	1.43	1.13	3.29	3.11
CAT3	2.93	2.60	2.91	4.45	3.94

**Table 2:** Group characteristics.

	Age	Height	Weight	Fat Mass %
N	20	20	20	20
Missing	0	0	0	0
Mean	16.4	171	62.2	8.47
Median	16.4	171	61.4	8.50
Standard deviation	0.3	3.0	3.7	0.6
Minimum	16.0	165	57.0	7.40
Maximum	16.9	176	69.1	9.40

**Table 3:** Typical training microcycle.

GD+1	GD+2	GD-4	GD-3	GD-2	GD-1
Day Off	Dynamic warm-up 10′	Dynamic warm-up 10′	Dynamic warm-up 10′	Dynamic warm-up 10′	Dynamic warm-up 10′
	Resistance training targeting hypertrophy or max strength 30′	Possession game 8 vs. 8–10 vs. 10 12′	Possession game 4 vs. 4 + 3 joker in reduced space 25 × 18 m 12′	Passing drills 12′	Coordination, acceleration, reaction 10′
	Tactical game 10 vs. 10 + 2 GK in reduced space 60 × 50 m 15′	Tactical content: game in large space 94 × 60 m targeting transition 20′	Tactical content: game 9 vs. 9 in reduced space 55 × 45 m targeting pressing 15′	Tactical content 20′	Tactical content 20′
		Large-sided game: 10 vs. 10 + 2 GK 15′	Small-sided games: 3 vs. 3–5 vs. 5 + 2 GK 12′–15′	Game 10 vs. 10 + 2 GK in reduced space 60 × 50 m 12′	Game 10 vs. 10 + 2 GK in reduced space 70 × 50 m 10′
					Set pieces offensive/defensive 15′

**Table 4:** Correlation between weekly training and game running performance variables.

	df	Total Distance	HSR Distance	ZONE6 Distance	ACC > +2 m/s^2^	DEC > −2 m/s^2^
MIC1	26	0.788 ***	0.701 ***	0.572 ***	0.249	0.949 ***
MIC2	30	0.756 ***	0.278	0.270	0.472 **	0.460 **
MIC3	17	0.858 ***	0.892 ***	0.547 *	0.601 **	0.569 *
MIC4	27	0.880 ***	0.781 ***	0.481 **	0.432 *	0.449 *
MIC5	25	0.880 ***	0.781 ***	0.455 *	0.432 *	0.449 *
MIC6	27	0.800 ***	0.393 *	0.383 *	0.357	0.347
MIC7	30	0.740 ***	0.270	0.096	0.397 *	0.637 ***
MIC8	30	0.848 ***	0.512 **	0.258	0.190	0.382 *
MIC9	28	0.821 ***	0.814 ***	0.563 ***	0.638 ***	0.684 ***
MIC10	22	0.871 ***	0.705 ***	0.542 **	0.457 *	0.478 *
MIC11	22	0.924 ***	0.812 ***	0.659 ***	0.675 ***	0.768 ***
MIC12	32	0.706 ***	0.805 ***	0.601 ***	0.270	0.387 *
MIC13	45	0.823 ***	0.815 ***	0.596 ***	0.362 *	0.461 **
MIC14	30	0.701 ***	0.634 ***	0.587 ***	0.467 **	0.486 **
MIC15	30	0.894 ***	0.847 ***	0.564 ***	0.464 **	0.490 **
MIC16	40	0.796 ***	0.608 ***	0.275	0.274	0.576 ***
MIC17	32	0.836 ***	0.815 ***	0.492 **	−0.059	0.086
MIC18	33	0.905 ***	0.749 ***	0.682 ***	0.573 ***	0.698 ***

*** Correlation is significant at the 0.001 level (2-tailed). ** Correlation is significant at the 0.01 level (2-tailed). * Correlation is significant at the 0.05 level (2-tailed).

## Data Availability

Data are available upon request from the corresponding author.
